# Antiamoebic Activity of *Adenophyllum aurantium* (L.) Strother and Its Effect on the Actin Cytoskeleton of *Entamoeba histolytica*

**DOI:** 10.3389/fphar.2016.00169

**Published:** 2016-06-27

**Authors:** Mayra Herrera-Martínez, Verónica I. Hernández-Ramírez, Beatriz Hernández-Carlos, Bibiana Chávez-Munguía, Mónica A. Calderón-Oropeza, Patricia Talamás-Rohana

**Affiliations:** ^1^Departamento de Infectómica y Patogénesis Molecular, Centro de Investigación y de Estudios Avanzados del Instituto Politécnico NacionalCiudad de México, Mexico; ^2^Instituto de Agroindustrias, Universidad Tecnológica de la MixtecaHuajuapan de León, Mexico; ^3^Instituto de Genética, Universidad del MarSan Pedro Pochutla, Mexico

**Keywords:** actin, cytoskeleton, *Adenophyllum aurantium*, amoebiasis, *Entamoeba histolytica*, encystation

## Abstract

In Mexico, the *Adenophyllum aurantium* (L.) Strother plant is consumed as an infusion to treat intestinal diseases such as amoebiasis, which is an endemic health problem in Mexico and other countries. However, the effect of *A. aurantium* on *Entamoeba histolytica*, the causative agent of amoebiasis, is unknown. An aerial part methanolic extract (*Aa*MeA), a root methanolic extract (*Aa*MeR) and a root ethyl acetate extract (*Aa*EaR) were tested on *E. histolytica* trophozoites. *Aa*MeA and *Aa*MeR did not show antiproliferative activity; however, *Aa*EaR exhibited an *in vitro* GI_50_ of 230 μg/ml, and it was able to inhibit the differentiation of *Entamoeba invadens* trophozoites into cysts. The intraperitoneal administration of *Aa*EaR (2.5 or 5 mg) to hamsters that were infected with *E. histolytica* inhibited the development of amoebic liver abscesses in 48.5 or 89.0% of the animals, respectively. Adhesion to fibronectin and erythrophagocytosis were 28.7 and 37.5% inhibited by *Aa*EaR, respectively. An ultrastructure analysis of *Aa*EaR-treated trophozoites shows a decrease in the number of vacuoles but no apparent cell damage. Moreover, this extract affected the actin cytoskeleton structuration, and it prevented the formation of contractile rings by mechanism(s) that were independent of reactive oxygen species and RhoA activation pathways. ^13^C NMR data showed that the major compounds in the *Aa*EaR extract are thiophenes. Our results suggest that *Aa*EaR may be effective in treatments against amoebiasis, nevertheless, detailed toxicity studies on thiophenes, contained in *Aa*EaR, are required to avoid misuse of this vegetal species.

## Introduction

*Aa*EaR, ethyl acetate extract of *Adenophyllum aurantium* Root; ALA, amoebic liver abscess; *Aa*MeA, methanol extract of *Adenophyllum aurantium* aerial part; *Aa*MeR, methanol extract of *Adenophyllum aurantium* Root; DMSO, dimethyl sulfoxide; GI_50,_ concentration that causes the 50% maximal inhibition of cell proliferation; MTZ, metronidazole; ROS, reactive oxygen species; TEM, transmission electron microscopy. Amoebiasis is the third-most common cause of death from parasitic infections, and it has a very high incidence in countries such as India, Mexico, Central and South America, and others. *Entamoeba histolytica* is a protozoan parasite and the causative agent of this disease, which is associated with intestinal and extra-intestinal clinical manifestations. This amoeba has a two-stage life cycle, namely, the cyst, which is the infective stage, and the trophozoite, which is the invasive phase. The infection is acquired through food and water that has been contaminated with *E. histolytica* cysts (Lejeune et al., 2009). These cysts release amoebic trophozoites that may invade the intestinal mucosa and disseminate through a hematogenous route to other organs, primarily the liver, where the parasite usually causes amoebic liver abscess (ALA) formation (Centers for Disease Control and prevention [CDC], 2015). Globally, 50 million people suffer from amebic colitis and/or liver abscesses, which result in 50,000–100,000 deaths each year (Tanyuksel and Petri, 2003). Although, metronidazole is an effective treatment for invasive amoebiasis, side effects have been described in patients who were treated with this antiamoebic agent, and drug resistance has also been reported (Hanna et al., 2000; Upcroft and Upcroft, 2001; Bansal et al., 2004).

The development of novel and effective antiamoebic drugs that produce fewer or no side effects is urgently needed. Medicinal plants have an advantage in this area on the basis of their long term use by humans. The bioactive compounds obtained from these plants are expected to have low human toxicity (Fabricant and Farnsworth, 2001). In fact, natural products and their derivatives are estimated to represent approximately 50% of all drugs that have entered the market during the last three decades (Newman and Cragg, 2012).

Mexico is home to approximately 10% of the flora species in the world, with an estimated 23,000–25,000 higher plants species; 10% of them have a medicinal use (Magaña and Villaseñor, 2002). Therefore, Mexican plants represent a great potential source of new drugs. New molecules with antiamoebic activity have been isolated from the Asteraceae family (Calzada et al., 2009). *Adenophyllum aurantium* (L.) Strother is an endemic herb in Mexico (Villarreal and Villaseñor, 2004) of the Asteraceae family that is traditionally known in Mexico as “arnica silvestre.” Synonyms include *Aster aurantius* L., *Clappia aurantiaca* (L.) Benth., *Clomenocoma aurantia* (L.) Cass., *Dyssodia aurantia* (L.) B. L. Rob., *Inula aurantiaca* (L.) Spreng., and *Tagetes punctate* Sessé & Moc^1^. Antimicrobial activity from this plant has been reported against *Micrococcus luteus*, *Bacillus cereus, Staphylococcus epidermidis, Pseudomonas aeruginosa, Alternaria alternata*, and *Fusarium solani*; it is also reportedly innocuous toward cell lines such as KB (human nasopharyngeal carcinoma), Caco-2 (human colon carcinoma) and *Bacillus liqueniformis* (Frei et al., 1998; Lira-De León et al., 2014). Aerial parts of this vegetable species are used among Zapotec Indians (healers, midwives, and herbalists) to cure the stomachache (Frei et al., 1998). The bibliographic investigation carried out by Alonso-Castro et al. (2011) in Mexico scientific books, local encyclopedias of the Mexican Institute for Indigenous Studies (INI), and scientific published material on native Mexican medicinal flora showed that *A. aurantium* is used in Mexico to treat gastrointestinal disorders as diarrhea, dysentery, and gastric ulcers. Moreover, local undocumented sources (herbalists) from Puerto Escondido, Oaxaca, argue its use as anti-amoebic treatment (Catalina Gaspar Hernández, herbalist from this region, personal communication, 2014). Considering that *E. histolytica* produces symptoms such as those described above, we were interested in testing the anti-amoebic activity present in this plant.

In this study, we analyzed whether methanolic or ethyl acetate extracts of *A. aurantium* (aerial part or roots) were effective against *E. histolytica* trophozoites. The ethyl acetate extract was able to prevent different steps of the parasite’s pathogenic process such as: encystment, liver abscess development, fibronectin adhesion, and erythrophagocytosis. The extract’s effect on the actin cytoskeleton was analyzed as an approach to its mechanism of action.

## Materials and Methods

### Plant Extract

*Adenophyllum aurantium* was collected from the Chepilme Botanical Garden, Pochutla, Oaxaca, México, where *A. aurantium* is maintained and propagated for conservation, research, and educational purposes. A voucher specimen (65049) was deposited at the “Jorge Espinosa Salas” Herbarium in the Autonomous University of Chapingo, Texcoco, Mexico. The vegetal material was cleaned, dried and cut in pieces; then, the aerial parts (A) and roots (R) were macerated with methanol (Me) and left to stand for 2 weeks at room temperature (25°C), in the absence of light. The extract was passed through filter paper (No. 1 Whatman), and the vegetal residue was further macerated twice with the same amount of solvent, allowed to stand for 1 week more and then filtered. The filtrates that were obtained from each extraction were mixed and concentrated to dryness under a vacuum. A methanolic extract of the roots was further partitioned with AcOEt (Ea), and the solvent was eliminated. Each extract was dissolved in DMSO, and a stock concentration was kept at 4°C for further use.

Ethyl acetate extract (14.36 g) was subjected to column chromatography (SiO_2_) using different mixing ratios of hexane-ethyl acetate. Eluted fractions (with 94:6, 92:8, 90:10) yielded two pure compounds (**1, 2**) and a mixture of thiophene derivatives (**3**) [brown solids: (**1)** 23.7 mg (0.17%), (**2)** 33.9 mg (0.21%), and (**3)** 45 mg (0.31%) respectively].

(1) α-Terthienyl. ^1^H NMR (400 MHz, CDCl_3_) δ 7.173 (dd, *J* = 3.7, 1.2 Hz, H-3), 7.02 (dd, *J* = 5.1, 3.7 Hz, H-4), 7.22 (dd, *J* = 4.98, 1.17 Hz, H-5), 7.080 (s, H,3′, H-4′), 7.173 (dd, *J* = 3.66, 1.17 Hz, H-3′′), 7.021 (dd, *J* = 5.12, 3.66 Hz, H-4′′), 7.218 (dd, *J* = 4.98, 1.17 Hz, H-5′′). ^13^C NMR (100 MHz, CDCl_3_) δ 137.1 (C-2, C-5′′), 123.7 (C-3, C-4′′), 127.9 (C-4, C-4′′), 124.5 (C-5, C-2′′), 136.2 (C-2′, C-5′), 124.3 (C-3′, C-4′).
(2) 5-(4′′-Hydroxy-1′′-butynyl)-2-2′-bithiophene. ^1^H NMR (400 MHz, CDCl_3_) δ 7.05 (d, *J* = 3.8 Hz, H-3), 7.00 (d, *J* = 3.8 Hz, H-4), 7.16 dd (*J* = 3.6, 1.21 Hz, H-3′) 7.017 (d, *J* = 3.6 Hz, H-4′) 7.2 (dd, 5.16, 1.21 Hz, H-5′), 2.7 (dd, *J* = 6. 2, 6.1 Hz, H-3′′), 3.8 (t, *J* = 6.22 Hz, H-4′′). ^13^C NMR (100 MHz, CDCl_3_) δ 138-05 (C-2), 132.4 (C-3), 123.26 (C-4), 122.0 (C-5), 136.75 (C-2′), 124.1 (C-3′), 127.9 (C-4′), 124.8 (C-5′), 91.54 (C-1′′), 75.5 (C-2′′), 24.19 (C-3′′), 60.98 (C-4′′).

### Parasites

Trophozoites of the HM1-IMSS *E. histolytica* strain and IP-1 *Entamoeba invadens* strain (ATCC 30994) were axenically cultivated in TYI-S-33 medium that was supplemented with 10% (v/v) adult bovine serum and 1% (v/v) Diamond Vitamin-Tween 80 solution (JRH Biosciences, Lenexa, KS, USA) at 37 and at 26°C, respectively (Diamond et al., 1978).

### Measuring *In Vitro* Antiamoebic Activity by WST-1 Assay

Trophozoites (1 × 10^4^) were incubated with 200 μg/ml of each extract (aerial part methanolic extract, *Aa*MeA; root methanolic extract, *Aa*MeR; and root ethyl acetate extract, *Aa*EaR) for 48 h at 37°C. After the treatments, the live trophozoites were detached by chilling them on ice, and the number of live cells was determined by WST-1 method (05015944001, Roche Diagnostics, Meylan, France). The trophozoites were resuspended in 100 μl of PBS with 5 μl of WST-1 and incubated for 30 min. After the incubation period, absorbance measurements were performed in an ELISA Microplate Reader (Bio-Rad model 680) at 450 nm. A calibration curve for the cell number relative to the absorbance was used to determine the number of trophozoites. The growth inhibition (GI) percentage was obtained by comparing the results with those of untreated amoebas (control) with the following formula: %GI = (1-[Extract/Control]) × 100.

To analyze if there was a dose-response correlation with the *Aa*EaR treatment, trophozoites (1 × 10^4^) were incubated for 48 h at 37°C in the presence of different *Aa*EaR concentrations, starting with 400 μg/ml and going down to 25 μg/ml via two serial dilutions. DMSO (as vehicle) was used as the negative control and metronidazole (0.25 μg/ml) was used as the positive control in all the assays. An analysis of the proliferation rate was performed by incubating the trophozoites (1 × 10^4^) with *Aa*EaR at different times, and the cell number was determined at 12, 24, 48, and 72 h. To analyze if amoebas could recover after extract treatment, *Aa*EaR-treated trophozoites, over the course of 48 h, were washed twice with TYI-S-33 to eliminate the extract completely, and the amoebas (1 × 10^4^) were then incubated for 72 h in TYI-S-33 without treatment; the cell number was then determined. The GI_50_ (GI 50, or the concentration that caused 50% maximal inhibition of cell proliferation) values were calculated by linear regression analysis with the fitted line. The results were analyzed with GraphPad Prism 5.0 software.

### *Entamoeba invadens* Encystation

*Entamoeba invadens* trophozoites that were harvested during the logarithmic growth phase (5 × 10^5^/ml) were transferred into induction medium, 47% LG (TYI without glucose) with 5% adult bovine serum (Sánchez et al., 1994). The cultures were incubated at 26°C in the presence of their respective treatments, and after 72 h, the cells were counted with a hemocytometer to establish the number of trophozoites, round precysts (20–40 μm), and cysts (10–20 μm). The cell percentage was obtained by comparing the treated and untreated cultures (control) with the following formula: % Cell type = (Cell number with treatment/total cell number in control) x 100. The cells were fixed with 4% ρ-formaldehyde and stained with Calcofluor White M2R (0.02%; E6259, Sigma-Aldrich, St. Louis, MO, USA) for 1 h. The samples were observed under a Nikon ECLIPSE 80i fluorescence microscope (Nikon, Tokyo, Japan), and micrographs of each condition were obtained.

### *In Vivo* Antiamoebic Activity

The research protocol was approved by CINVESTAV’s Institutional Animal Care and Use Committee (CINVESTAV-IACUC). Experiments were performed with animals provided by Animal Production and Experimentation Unit (UPEAL-CINVESTAV) following the specifications of the Mexican National Norm (NOM-062-ZOO-1999) that is a version of the guide for the care and use of laboratory animals 2011. Male hamsters (*Mesocricetus auratus*) weighing approximately 80–100 g were intrahepatically inoculated with 1.5 × 10^6^ amoebas to induce ALA development (Tsutsumi et al., 1984). After 4 days post-infection, the *Aa*EaR (2.5 or 5 mg/hamster), MTZ (0.1 mg) and vehicle were administered daily by intraperitoneal route for the 4 days to follow, and then the hamsters were sacrificed. Their livers were dissected and weighed before and after the amoebic abscesses were removed to obtain the ALA percentage. Four animals were used per condition per experiment in two independent experiments.

### Adhesion to Fibronectin (FN) Assays

After being treated with *Aa*EaR (230 μg/ml) for 48 h, the trophozoites (2 × 10^5^) were washed in TYI-S-33 without bovine serum to eliminate the drug residues, and then the cells were incubated in TYI-S-33 with bovine serum for 10 min in purified human fibronectin-coated (100 μg) 24-well plates at 37°C (Talamás-Rohana et al., 1992). Non-adhered cells were eliminated, and adhered cells were detached by chilling and counted with the help of Trypan blue.

### Erythrophagocytosis Assays

After 48 h of *Aa*EaR (230 μg/ml) treatment, the trophozoites (2 × 10^5^) were washed in TYI-S-33 without bovine serum to eliminate the drug residues. Erythrocytes were then added (1:50 ratio), and the interaction was allowed to proceed for 10 min at 37°C in TYI-S-33 medium. Erythrophagocytosis was measured by two assays; in the first, the cells were fixed with 2.5% glutaraldehyde and analyzed by phase-contrast microscopy (Voigt et al., 1999). In the second assay, and for a quantitative analysis, non-fixed trophozoites were washed with water to eliminate the non-ingested erythrocytes. The trophozoites were then lysed with formic acid, and the amount of hemoglobin was measured by spectrophotometric analysis at 400 nm. The erythrophagocytosis percentage was obtained in relation to untreated amoebas.

### Transmission Electron Microscopy

The samples were fixed in 2.5% (v/v) glutaraldehyde in 0.1 M sodium cacodylate buffer pH 7.2 for 60 min. They were then post-fixed for 60 min with 1% (w/v) osmium tetroxide in the same buffer. After they were dehydrated in increasing concentrations of ethanol and propylene oxide, the samples were embedded in Polybed epoxy resins and polymerized at 60°C for 24 h. Thin sections (60 nm) were contrasted with uranyl acetate and lead citrate before being examined in a Jeol JEM-1011 electron microscope (Jeol, Ltd., Tokyo, Japan).

### Fluorescence Microscopy

Amoebas (2 × 10^5^) were adhered to coverslips that had been previously covered with 100 μg of purified human fibronectin for 15 min; they were then treated with *Aa*EaR (230 μg/ml) for 1 h. Bound cells were fixed with 4% ρ-formaldehyde for 1 h at 37°C and stained with rhodamine phalloidin (1:50; R415, Invitrogen, Carlsbad, CA, USA) for 1 h at 37°C. The samples were mounted with Vecta-Shield medium (Vector Laboratories, Peterborough, UK) and observed under a Carl Zeiss LSM 700 confocal microscope (Carl Zeiss, Jena, Germany).

### DCF-DA Cellular Reactive Oxygen Species Detection Assay

Trophozoites (5 × 10^5^) were treated with *Aa*EaR (230 μg/ml), with H_2_O_2_ (400 μM) as a positive control for ROS production and DMSO as a negative control for 1 h. After the incubation period, the samples were washed twice with PBS. The cells were resuspended in PBS with DCF-DA (25 μM; ms958, MitoSciences, Eugene, OR, USA), incubated for 15 min at 37°C, and then lysed. Their fluorescence was measured on black plates with a Microplate Luminescence Reader (Fluoroskan Ascent FL, Thermo, Boston, MA, USA).

### RhoA, Rac1, 2, 3, and Cdc42 Activity Measurement

The RhoA, Rac1, 2, 3, and Cdc42 activities were determined with luminescence or colorimetry-based G-LISAs (BK121, BK125, and BK127, respectively; Activation Assay Biochem Kits G-LISA Cytoskeleton, Inc., Denver, CO, USA) according to the manufacturer’s instructions. Proteins were obtained by incubating the cells with the provided cell lysis buffer, which contained protease inhibitors, and the cell extracts were immediately clarified by centrifugation at 18,000 × *g* at 4°C for 2 min. The protein concentrations were determined according to the manufacturer’s protocol, and the cell extracts were equalized to a protein concentration of 1 mg/ml for the respective assays. The samples were dispensed into the wells of the G-LISAs plates, which were coated with active GTPase-binding protein. The plates were placed on a cold microplate shaker set at 400 rpm for 30 min (RhoA and Rac) and for 15 min (Cdc42) at 4°C. The plates were then washed three times with Wash Buffer at room temperature. Anti-GTPase primary antibodies (at recommended dilutions) were added to each well, and the plates were placed on the shaker for 45 min (RhoA and Rac) and for 30 min (Cdc42). After three washes, the plates were incubated with secondary antibodies for 45 min (RhoA and Rac) and for 30 min (Cdc42), and the HRP detection reagent (as provided in the enhanced chemiluminescence kit) was added to the wells. Active RhoA was detected by tracking the luminescence signal with a Microplate Luminescence Reader (Fluoroskan Ascent FL, Thermo, Boston, MA, USA); Rac1, 2, 3, and Cdc42 were detected by colorimetric analyses in an ELISA microplate reader (Model 680, BioRad, Hercules, CA, USA). A calibration curve with a positive control for each enzyme was performed, and the results are shown as ρg of GTPase-GTP/μg protein extract. For RhoA, the results are representative of two independent experiments that were performed in duplicate; for Rac1, 2, 3, and Cdc42, only one experiment was performed in duplicate.

### Nuclear Magnetic Resonance Spectroscopy

Nuclear magnetic resonance measurements were performed with 5 mm probes at 22°C from methanol-d (CD_3_OD) or dimethylsulfoxide-d6 (DMSO-d6) solutions, with 0.03% tetramethylsilane (TMS) as the internal standard, on a Bruker ASCEND TM 400 (400 MHz proton frequency) spectrometer (Bruker, Rheinstetten, Germany) at 298 K. Chemical shifts (δ) from TMS are reported in ppm.

### Statistical Analysis

The data are representative of three independent experiments, with each one in duplicate except for the Rac 1, 2, 3, and cdc42 experiments. A statistical analysis was performed with GraphPad Prism 5.0 software (GraphPad Software, San Diego, CA, USA), and a Student’s *t*-test was used to determine the *p*-values for the differences that were observed between the tested compounds and the control. One-way ANOVA analysis with a Bonferroni post-test was applied to the following assays: different doses of the plant extract, different doses of metronidazole, and *in vivo* assays. Mean is presented and error bars represent standard deviation (SD). The results were considered significantly different when ^∗^*p* < 0.05, ^∗∗^*p* < 0.01, and ^∗∗∗^*p* < 0.001.

## Results

### *In Vitro* Antiproliferative Activity of *Aa*EaR Extract

**FIGURE 1:**
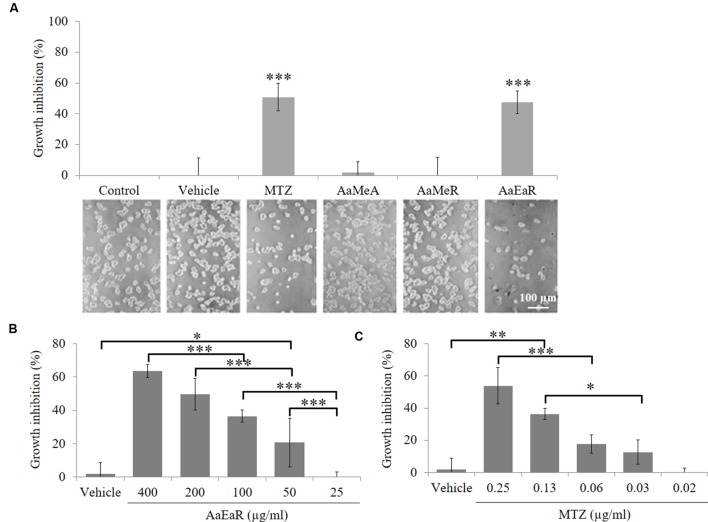
***In vitro* activity of *Aa*EaR against *Entamoeba histolytica* trophozoites**. The trophozoites (1 × 10^4^) were treated (200 μg/ml) with aerial part methanolic extract (*Aa*MeA), root methanolic extract (*Aa*MeR), root ethyl acetate extract (*Aa*EaR) and MTZ (0.25 μg/ml) for 48 h to obtain the % of growth inhibition by using the formula (1-[Extract/Control]) × 100. The figure shows the graphical and corresponding images of the different treatments **(A)**. The trophozoites (1 × 10^4^) were treated with *Aa*EaR **(B)** or MTZ **(C)** at different concentrations. The data are representative of three independent experiments that were performed in duplicate, error bars represent SD. ANOVA analysis was used to compare among treatments **(B,C)**. ^∗^*p* < 0.05, ^∗∗^*p* < 0.01, ^∗∗∗^*p* < 0.001.

The use of *A. aurantium* to treat intestinal diseases suggested that it could have antiamoebic activity. Lira-De León et al. (2014) previously suggested that ethyl acetate extract from the root was more active than the methanolic extract from the aerial part against fungi. In this work, we decide to analyze the effect on the proliferation of *E. histolytica* trophozoites, of a methanolic extract of the aerial part (*Aa*MeA), a methanolic extract of the root (*Aa*MeR) and an ethyl acetate extract of the root (*Aa*EaR), all of them at 200 μg/ml. The methanolic extracts (*Aa*MeA and *Aa*MeR) did not have antiproliferative activity against *E. histolytica*, but *Aa*EaR inhibited the proliferation by almost 50% (**Figure 1A**). To obtain the GI_50_ (the GI 50, or the concentration that caused the 50% maximal cell proliferation inhibition), different concentrations of *Aa*EaR (**Figure 1B**) and MTZ (**Figure 1C**) were evaluated. *Aa*EaR concentrations above 400 μg/ml were not tested because of the extract’s solubility characteristics. The *Aa*EaR extract had a dose-dependent effect, in which 400 μg/ml caused the highest inhibition (63.56 ± 3.85%) against trophozoite proliferation (**Figure 1B**). The *Aa*EaR GI_50_ was 230.18 μg/ml, and the MTZ GI_50_ was 0.25 μg/ml (**Table 1**).

**Table 1:** *Adenophyllum aurantium* extract and metronidazole GI_50_ values against *Entamoeba histolytica* trophozoites.

Treatment	^a^GI_50_ (μg/ml)
*Aa*EaR	^b^230.18 ± 19.55
Metronidazole	^c^0.25 ± 0.05

*^a^Three independent experiments were performed in duplicate (*n* = 6); ^b^*R*^2^ = 0.98; ^c^*R*^2^ = 0.97*.

### *Aa*EaR Inhibits Cyst Formation in *Entamoeba invadens*

**FIGURE 2:**
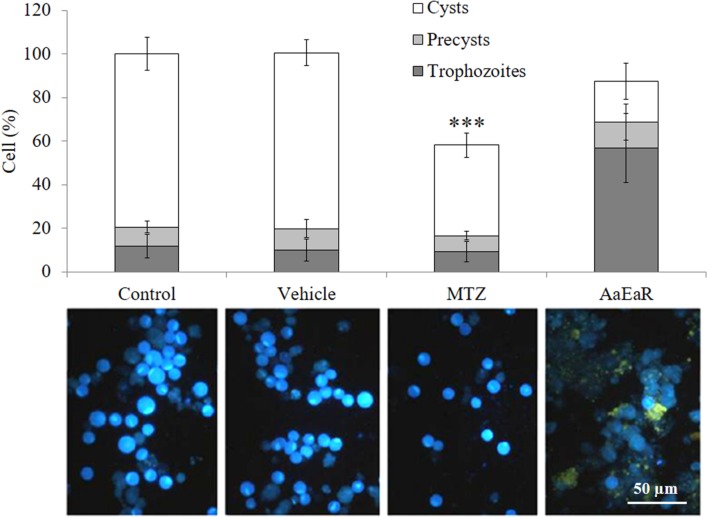
***Aa*EaR prevents the differentiation of *Entamoeba invadens***. *E. invadens* trophozoites were induced to encyst in LG medium diluted at 47% with 5% ABS, in the presence of *Aa*EaR (230 μg/ml) for 72 h. The trophozoites, precysts and cysts were counted, and the cell percentage was obtained using 100% as the total cell number under the control condition without treatment. Representative images of cells that were stained with Calcofluor White m2r are shown below the graph, and the cysts are more fluorescent than the trophozoites. Two independent experiments were performed in duplicate (*n* = 4), error bars represent SD. ^∗∗∗^*p* < 0.001.

During a non-invasive *E. histolytica* infection, individuals become asymptomatic carriers and pass cyst in their stools. Therefore, it is very important to find drugs that are able to inhibit cyst formation and thereby block the transmission of the parasite. However, the experimental encystment of *E. histolytica* has not been successful yet, and for this reason, *E. invadens* is being used as a model to study encystation. In addition, this parasite presents morphological and genetic characteristics that are similar to those of *E. histolytica*, and its encystment is easily induced *in vitro*. Thus, the effect of *Aa*EaR on *E. invadens* cyst formation was evaluated. Trophozoites, precysts and cyst percentages were determined after treating the amoebas with MTZ or *Aa*EaR, and by using as 100% the total cell number in the control condition. The *Aa*EaR treatment decreased cyst formation, and it exhibited a higher percentage of trophozoites and a lower percentage of cysts in comparison with the control (**Figure 2**), suggesting that *Aa*EaR prevented trophozoite-to-cyst differentiation. By contrast, MTZ decreased the cyst percentage to 41% and the final number of cells by 58%, suggesting that MTZ killed trophozoites before they underwent differentiation to become cysts.

### Intraperitoneal Administration of *Aa*EaR Inhibits the Development of Amoebic Liver Abscesses (ALAs) in Hamsters

**FIGURE 3:**
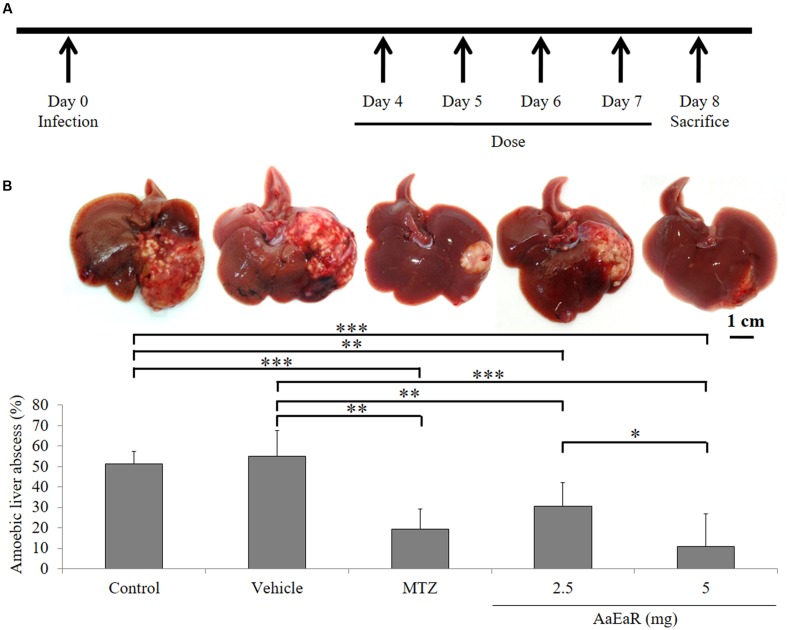
***Aa*EaR inhibits the development of amebic liver abscesses**. Trophozoites (1.5 × 10^6^) were inoculated in hamsters, and 4 days post-infection, animals were treated over the 4 days with vehicle, MTZ (0.1 mg) or *Aa*EaR (2.5 and 5 mg) by intraperitoneal route **(A)**. Representative images and graphics are shown **(B)**. Two independent experiments were performed using four animals per experiment, error bars represent SD. ANOVA analysis was used to compare among treatments. ^∗^*p* < 0.05, ^∗∗^*p* < 0.01, ^∗∗∗^*p* < 0.001 (*n* = 8).

Having established the *in vitro* effect of the *Aa*EaR extract, we then analyzed whether the extract could affect the development of ALAs in the hamster model. The hamsters were inoculated intrahepatically with 1.5 × 10^6^ amoebas, and from days 4 to 7 post-infection, 2.5 or 5 mg of *Aa*EaR or 0.1 mg of MTZ were administered by intraperitoneal route. The animals were sacrificed at day 8 (**Figure 3A**). Untreated or vehicle-treated animals developed abscesses (51.37 ± 6.18% and 55.23 ± 12.48%, respectively) as well as multiple granulomas (**Figure 3B**), and animals that were treated with *Aa*EaR (2.5 and 5 mg) or MTZ (0.1 mg) developed smaller-sized ALA, at 30.81 ± 11.43%, 10.98 ± 16.07%, and 19.35 ± 9.96%, respectively (**Figure 3B**). Thus, MTZ and *Aa*EaR treatments (2.5 and 5 mg) decreased the ALA development by ^∗∗∗^62.32, ^∗∗^46.56, and ^∗∗∗^78.62%, respectively, in comparison with control condition (^∗∗∗^*p* < 0.001 and ^∗∗^*p* < 0.01, *n* = 8).

### *Aa*EaR Treatment Affects Adhesion to Fibronectin and Erythrophagocytosis

**FIGURE 4:**
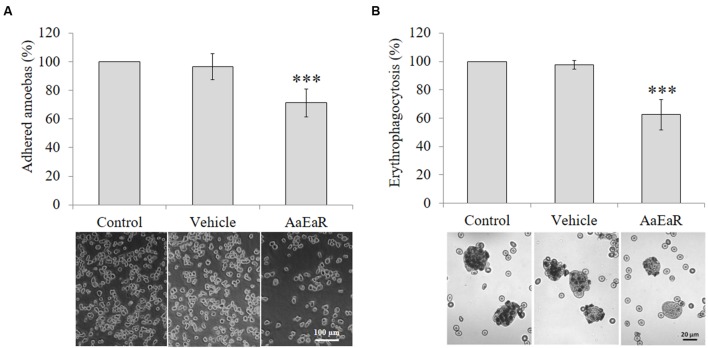
***Aa*EaR affects adhesion and erythrophagocytosis in *E. histolytica***. The trophozoites (2 × 10^5^) were treated with *Aa*EaR (230 μg/ml) for 48 h. The treated cells were incubated on fibronectin-coated coverslips (100 μg/ml) for 15 min and the adhered amoebas were counted **(A)**. The amoebas were incubated with erythrocytes (1:50 ratio) for 15 min, and after washing off the non-ingested erythrocytes, the total hemoglobin content was measured **(B)**. The data are representative of three independent experiments that were performed in duplicate, error bars represent SD. ^∗∗∗^*p* < 0.001.

Extracellular matrix degradation and erythrophagocytosis are among the virulence markers of pathogenic *E. histolytica* (Talamás-Rohana and Meza, 1988; Talamás-Lara et al., 2014). Thus, *Aa*EaR was evaluated to analyze its effect on fibronectin adhesion and erythrophagocytosis. *Aa*EaR decreased 28.67% the adhesion to fibronectin (**Figure 4A**) and 46.11% the erythrophagocytosis (**Figure 4B**).

### *Aa*EaR Treatment Decreases the Vacuole Numbers in *E. histolytica* Trophozoites

**FIGURE 5:**
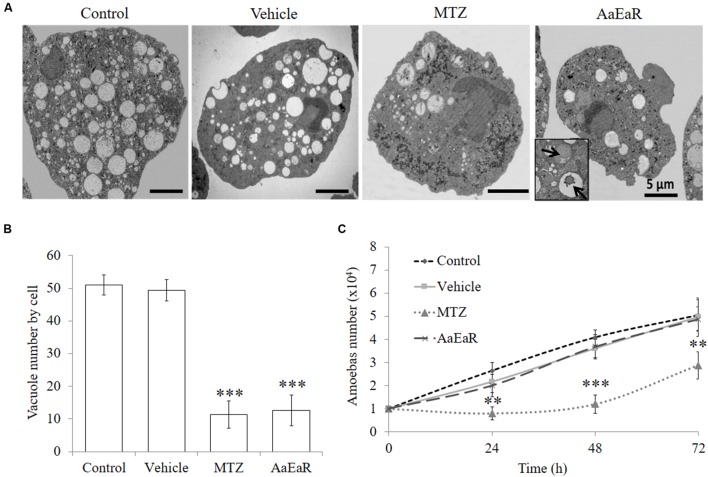
***Aa*EaR does not induce cell death characteristics**. The trophozoites (1 × 10^4^) were treated with *Aa*EaR (230 μg/ml) and MTZ (0.25 μg/ml) for 48 h. An ultrastructure analysis by transmission electron microscopy **(A)** and the number of vacuoles per cell are shown **(B)**. The data were collected from five images per condition. Amoebas (1 × 10^4^) were treated for 48 h with *Aa*EaR (230 μg/ml) or MTZ (0.25 μg/ml), washed and reincubated in fresh TYI-S-33 medium without treatment, and their proliferation was determined at different times **(C)**. The data are representative of three independent experiments that were performed in duplicate, error bars represent SD. ^∗∗^*p* < 0.01, ^∗∗∗^*p* < 0.001.

After we confirmed that *Aa*EaR had a negative effect on trophozoite proliferation, it was in our interest to analyze the mechanism of action by which this extract inhibits amoeba proliferation and the relevant pathogenicity processes in this parasite. The ultrastructure of treated trophozoites was analyzed by TEM by seeking evidence of morphological changes related to the *Aa*EaR treatment. The parasites that were incubated with MTZ displayed morphological alterations such as increased glycogen granules, a decrease in the number of vacuoles and nucleus damage in comparison with untreated and vehicle-treated trophozoites (**Figure 5A**). The *Aa*EaR-treated trophozoites maintained their nuclear and plasma membrane integrity but showed a significant reduction in their number of vacuoles (**Figure 5B**) as well as the presence of slightly electron-dense vacuoles (arrows in **Figure 5A**). To find whether the amoebas could recover after treatment, the trophozoites that had been treated with MTZ or *Aa*EaR for 48 h were incubated in fresh TYI-S-33 medium, and their proliferation was determined. *E. histolytica* proliferation was affected after being treated with MTZ but not with *Aa*EaR (**Figure 5C**).

### *Aa*EaR Modifies Contractile Ring Formation

**FIGURE 6:**
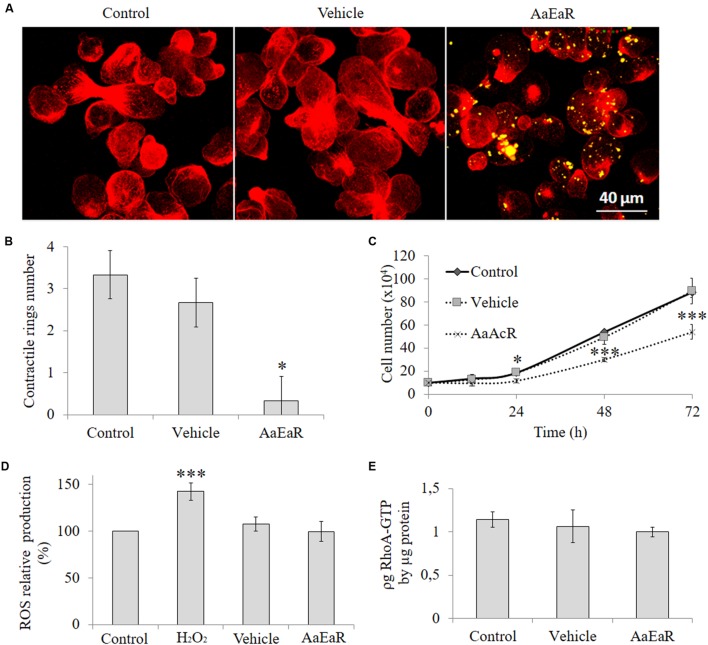
***Aa*EaR altered the formation of contractile rings in *E. histolytica* through reactive oxygen species and RhoA GTPase activity independent pathways**. The trophozoites (2 × 10^5^) were treated with *Aa*EaR (230 μg/ml) for 1 h, and filamentous actin was stained with rhodamine phalloidin (red); and the *Aa*EaR extract exhibits auto-fluorescence (yellow; **A**). The contractile ring number was determined in 20 amoebas from three different optical fields with the ImageJ program **(B)**. The amoebas (2 × 10^5^) were incubated with *Aa*EaR (230 μg/ml) for 72 h and the cell number was determined at different times **(C)**. The ROS production was evaluated by DCF-DA test, and the percentage was obtained by comparing them with untreated amoebas **(D)**. The active RhoA was measured by G-LISA cytoskeleton kit **(E)**. The data are representative of two independent experiments that were performed in triplicate or duplicate, error bars represent SD. ^∗^*p* < 0.05, ^∗∗∗^*p* < 0.001.

*Aa*EaR extract inhibited cell proliferation, a process in which diverse molecular complexes can participate. This process is commanded at the structural level by the actin cytoskeleton, an essential component for processes such as adhesion, protease secretion, chemotaxis, phagocytosis, capping, and cytokinesis in *E. histolytica* (Meza et al., 2006; Chávez-Munguía et al., 2008). Given the above results, this organelle could be a target for the compound(s) that are present in the root extract. To see whether *Aa*EaR affected the actin cytoskeleton, trophozoites that were treated with *Aa*EaR (230 μg/ml) were analyzed by confocal microscopy, which uncovered an altered actin cytoskeleton (**Figure 6A**) with a decrease in the level of polymerized actin (red; Supplementary Figure S1) and in the number of contractile rings (**Figure 6B**) in comparison with the control cells. This extract also decreased the proliferation rate of *E. histolytica* trophozoites that were treated for 72 h (**Figure 6C**). Some of the extract compounds are autofluorescent and were found inside vacuoles (as yellow color due to autofluorescence in green and red channels) 1 h post-treatment (**Figure 6A**, right panel); vacuoles’ autofluorescence was also observed (purple color due to autofluorescence in red and blue channels) at 48 h (Supplementary Figure S2). However, we did not determine the type of vacuoles in which these compounds were internalized.

Reactive oxygen species reportedly affect the function of the actin cytoskeleton (Shahi et al., 2016). To analyze this finding, oxygen intermediates were measured with 2′,7′-dichloro fluorescin diacetate (DCF-DA), a fluorogenic dye that measures hydroxyl, peroxyl, and other ROS within the cell. Hydrogen peroxide was used as the positive control for ROS production, and it increased the amount of ROS; however, *Aa*EaR did not induce an increase in their level, which was the same as that of untreated and vehicle-treated trophozoites (**Figure 6D**). The GTPases of the Rho family also have an important role in a variety of biological processes that are associated with stress fiber formation in *Entamoeba* (Ríos et al., 2008); therefore, we evaluated if there were any changes in the levels of active RhoA, Rac1, 2, 3, and Cdc42 in the *Aa*EaR-treated amoebas. The RhoA-GTP levels were similar in *Aa*EaR-treated amoebas in comparison with vehicle-treated amoebas (**Figure 6E**). In addition, Rac1, 2, 3, and Cdc42 were also analyzed, and they did not experience changes in their active form levels (Supplementary Figure S3).

### Presence of Thiophenes in *Aa*EaR

**FIGURE 7:**
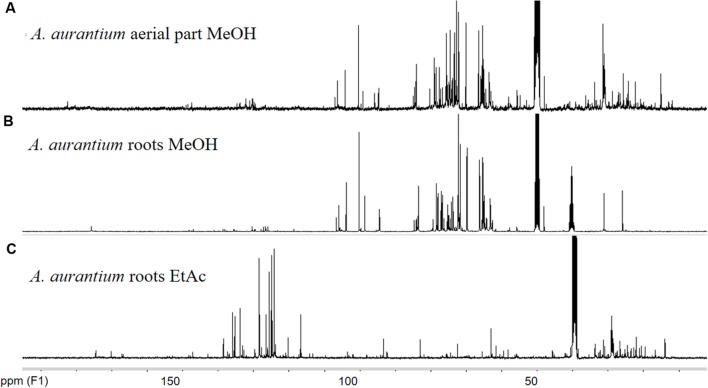
**^13^C NMR spectra of *Adenophyllum aurantium* extracts**. NMR measurements were performed in a Bruker ASCENDTM 400 (400 MHz proton frequency) spectrometer at 298 K. Chemical shifts (δ) are reported in ppm from tetramethylsilane (TMS). Solvents used were CD_3_OD **(A,B)** and DMSOd6 **(C)**.

**FIGURE 8:**
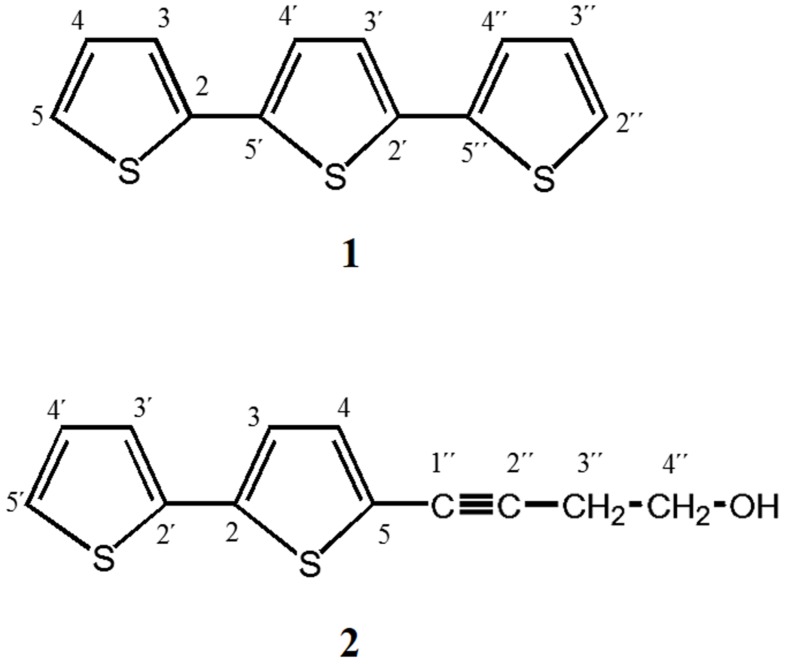
**α-Terthienyl and 5-(4′′-Hydroxy-1′′-butynyl)-2-2′- bithiophene were isolated from *Aa*EaR**. Ethyl acetate extract (14.36 g) was subjected to column chromatography (SiO_2_) different mixing ratios of hexane-ethyl acetate. Eluted fractions between 94:6 and 92:8 yielded the following brown solids: **(1)** α-Terthienyl, and **(2)** 5-(4′′-Hydroxy-1′′-butynyl)- 2-2′-bithiophene.

The increased effectiveness of pure compounds has been described for some medicinal plants (Alanís et al., 2003; Biswas et al., 2007; Ávila-Blanco et al., 2014; Mfotie Njoya et al., 2014). Thus, these results suggest that *Aa*EaR could possess active compounds with *in vitro* antiamoebic effectiveness, which correlates with its ethnopharmacological use against intestinal diseases. To investigate the chemical nature of the major compounds in the three extracts, ^13^C NMR data were obtained. Spectral profiles of *Aa*MeA and *Aa*MeR show the primary signals between δ 60 and 110, which were attributed to saccharides (Kazalaki et al., 2015; **Figures 7A,B**). In the spectrum for active *Aa*EaR extract, a reduced signal quantity was found between δ100 and 55, and the primary signals were between δ 150 and 110, and 50 and 15 (**Figure 7C**), the chemical shifts of which are characteristic of thiophenes and terpene-type compounds, respectively (Xi et al., 2014). This approximation was demonstrated by the isolation of two compounds (**1, 2**) and a mixture of thiophene derivatives (**3**) from *Aa*EaR extract. Compound **1** was identified as α-terthienyl on the basis of ^1^H and ^13^C NMR data (Xu et al., 2011; **Figure 8**). Compound **2** corresponded to 5-(4′′-hydroxy-1′′-butynyl)-2-2′-bithiophene according to ^1^H and ^13^C NMR data (Menelaou et al., 1991). The mixture (**3**) contains two thiophene derivates, which were observed from their ^13^C NMR data corresponding to acetylenic carbons (δ 96.1 and 95.5) and thiophene carbons (δ 123.3, 123.3, 123.9, 124.0, 124.6, 127.8, 127.9, 132.2, 132.3, 137.9, 138.1 data not shown; Takahashi et al., 2011).

## Discussion

The antiamoebic activity of ethyl acetate extract from *A. aurantium* was determined in this study. The GI_50_ of the antiamoebic extracts is classified as “good” if the samples displayed a GI_50_ of less than 20 μg/ml, “moderate” from 20 to 150 μg/ml and “weak” from 150 to 400 μg/ml; over 400 μg/ml is considered “inactive” (Calzada et al., 2009). According to this classification, *Aa*EaR (GI_50_: 230 μg/ml) could be classified as a weak antiamoebic extract; however, this particular classification was defined by using a small initial number of amoebas (6 × 10^3^) in comparison with the numbers used in this study (1 × 10^4^), and thus the capability of the extract may be underestimated. Even so, our results provide a basis for further research on the antiamoebic activity of *A. aurantium*.

The *Aa*EaR extract was evaluated during different steps of the *E. histolytica* pathogenic process. Parasitic differentiation from the trophozoite to the cyst was inhibited, and the impact of this finding could be encouraging from the perspective of decreasing the possible asymptomatic carriers that play an important role in the prevalence and incidence of this disease. A drug that is able to comply with these criteria could be relevant in controlling the transmission cycle of this parasite. However, the administration of *Aa*EaR to the *in vivo* model decreased ALA development, as did MTZ. Similar results with another plant extract have been reported for an essential oil (ascaridole) that was isolated from *Dysphania ambrosioides*, which contains the two amoebicidal compounds epoxi-ascaridole (45.5%) and *cis*-ascaridole (34.2%). These compounds can decrease ALA development in seven doses of 8 mg/day per animal (Ávila-Blanco et al., 2014).

Important processes in the pathogenicity of *E. histolytica* such as fibronectin adhesion and erythrophagocytosis were inhibited; these activities strongly depend on a functional actin cytoskeleton. Furthermore, the reduction in the number of vacuoles by *Aa*EaR as observed for other antiamoebics (Velázquez-Domínguez et al., 2013; Pais-Morales et al., 2016) implies that vesicular traffic might have been decreased or abolished. Vesicular traffic is a highly specialized function, and in *E. histolytica*, it is closely synchronized with the actin cytoskeleton organization. Our results confirm that the extract was able to affect the actin cytoskeleton rearrangement and that this process could be a target for the compounds present in the extract. The formation of contractile rings in *E. histolytica* was affected by *Aa*EaR, suggesting that cytokinesis could be inhibited, causing a decrease in the proliferation rate of *E. histolytica*. However, neither ROS production nor the GTPases of the RhoA family were affected by *Aa*EaR. These results suggest that there must be additional mechanisms that affect the actin cytoskeleton organization, possibly through direct binding to actin or to other structural components of the cytoskeleton. It is possible that RhoA activation does not play a role in *E. histolytica* cytokinesis as reported for other cells (Yoshizaki et al., 2003). Natural compounds that are derived from medicinal plants such as epichatechin and kaempferol have been shown to deregulate proteins related to the actin cytoskeleton (Bolaños et al., 2014, 2015).

The *Adenophyllum* genus belongs to the Pectidinae subtribe (Asteraceae), which has been shown to contain thiophenes (Downum et al., 1985). NMR data showed the presence of thiophenes in active *Adenophyllum* (*Aa*EaR) extract but not in inactive extract (*Aa*MeA and *Aa*MeR) against *E. histolytica* trophozoites. We isolated two thiophenes from *Aa*EaR extract: α-terthienyl and 5-(4′′-hydroxy-1′′-butynyl)-2-2′-bithiophene. It is possible that thiophenes are responsible for the presence of the slightly electron-dense vacuoles that were observed by TEM. Alpha-terthienyl is able to reduce the growth rate of *Microsporum cookie*, and it induces the presence of electron-dense vacuoles without causing damage to the cells (Mares et al., 1990). Thiophenes could also be the cause of autofluorescent vacuoles in *Aa*EaR-treated amoebas because these compounds are strongly fluorescent (Zhao et al., 2011).

The traditional use of *A. aurantium* indicates that people should not expose themselves to sunlight after drinking this plant infusion. This ethnomedical knowledge could be based on the harmful effects of α-terthienyl that occur under sunlight because this compound has photobiocidal effects that are associated with the presence of ultraviolet light (Kagan et al., 1984; Nivsarkar et al., 1991, 1996). Insect Tn-5B1-4 cells were more susceptible to the action of photoactivated alpha-terthienyl in comparison with human 293 cells, suggesting considerable differences between pathogen and mammal sensitivity to this compound (Huang et al., 2010). Oral administration of alpha-terthienyl in rats, at a single dose of 50 mg/kg, produced maximal excretion of the labeled material in 1 day after administration, and concentrations declined to no measurable quantities by day 4; intraperitoneal administration to rats showed an LD_50_ of 110 mg/kg (Marles et al., 1995). Nevertheless, more detailed studies showed that alpha-terthienyl could be a potential factor in human mutagenic progression (Huang et al., 2010). Drug metabolism of thiophene containing substrates by cytochrome P450s (CYP450) leads to toxic side effects, for example, nephrotoxicity (suprofen, ticlopidine), hepatotoxicity (tienilic acid), thrombotic thrombocytopenic purpura (clopidogrel), and aplastic anemia (ticlopidine; Jaladanki et al., 2015). The biological chemical study of plant species used in traditional medicine is a necessary task in order to confirm the safe use and the biological activity attributed. The scope of this work was to identify antiamoebic extracts or compounds from *A. aurantium*; however, the presence of thiophenes was not expected. Therefore, knowledge obtained from previous work in the literature provide the basis about probable risks of using this species in the treatment of amoebiasis.

## Conclusion

The *A. aurantium* extract was effective against *E. histolytica* trophozoites and was able to prevent different steps of this parasite’s pathogenic process, namely, encystment, liver abscess development, fibronectin adhesion and erythrophagocytosis. We suggest that the activity of the extract is related to its effect on the actin cytoskeleton rearrangement of *E. histolytica*, via signaling pathways independent of ROS and RhoA GTPase activity. Two thiophenes were identified in the active extract, where one of them is known for its toxicity. Based on these results, we propose going further in the isolation of all bioactive compounds from this vegetal species to identify the ones that are responsible for the observed anti-amoebic effects. In the meantime, the proposal to use this vegetal species to treat amoebiasis should be taken cautiously.

## Author Contributions

Acquisition of biological data: MH-M. Acquisition, analysis, and interpretation of chemical data: BH-C, MC-O. Transmission electron microscopy analysis: BC-M. Analysis and interpretation of data: MH-M, VH-R, and PT-R. Drafting of the manuscript: MH-M and PT-R. Provided reagents/materials/analysis tools: BH-C, BC-M, and PT-R.

## Conflict of Interest Statement

The authors declare that the research was conducted in the absence of any commercial or financial relationships that could be construed as a potential conflict of interest.

## Footnotes

1 http://www.theplantlist.org

## ABBREVIATIONS

*Aa*EaR: ethyl acetate extract of *Adenophyllum aurantium* Root
ALA: amoebic liver abscess
*Aa*MeA: methanol extract of *Adenophyllum aurantium* aerial part
*Aa*MeR: methanol extract of *Adenophyllum aurantium* Root
DMSO: dimethyl sulfoxide; GI_50,_ concentration that causes the 50% maximal inhibition of cell proliferation
MTZ: metronidazole
ROS: reactive oxygen species
TEM: transmission electron microscopy
