# Supplementary figures and images for: Antiamoebic Activity of Adenophyllum aurantium (L.) Strother and Its Effect on the Actin Cytoskeleton of Entamoeba histolytica
Source: Front Pharmacol. 2016 Jun 27;7:169. doi: 10.3389/fphar.2016.00169 (PMC4922267; doi:10.3389/fphar.2016.00169)

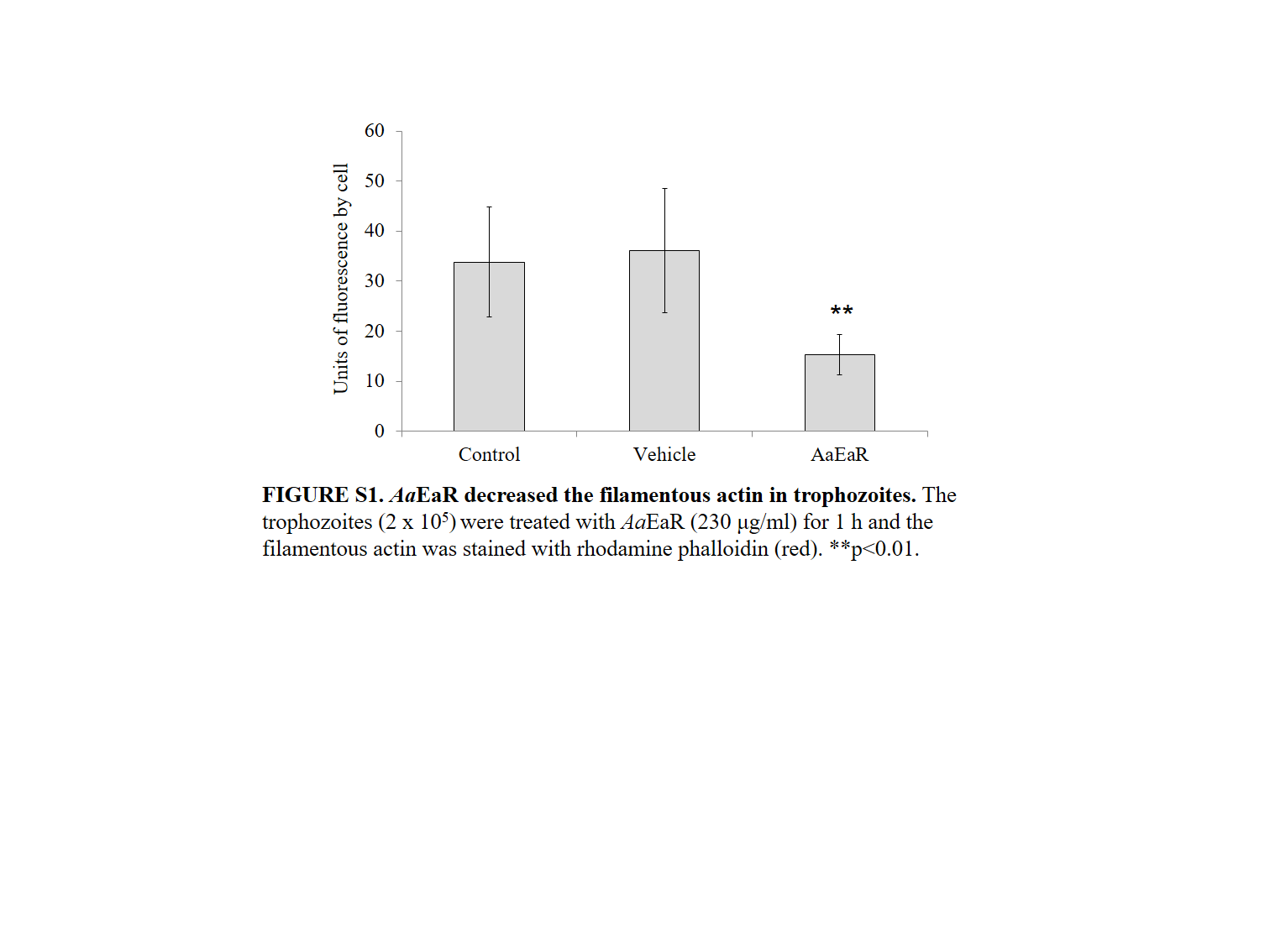

Supplement: Supplementary file 1 [file Image_1.TIF]

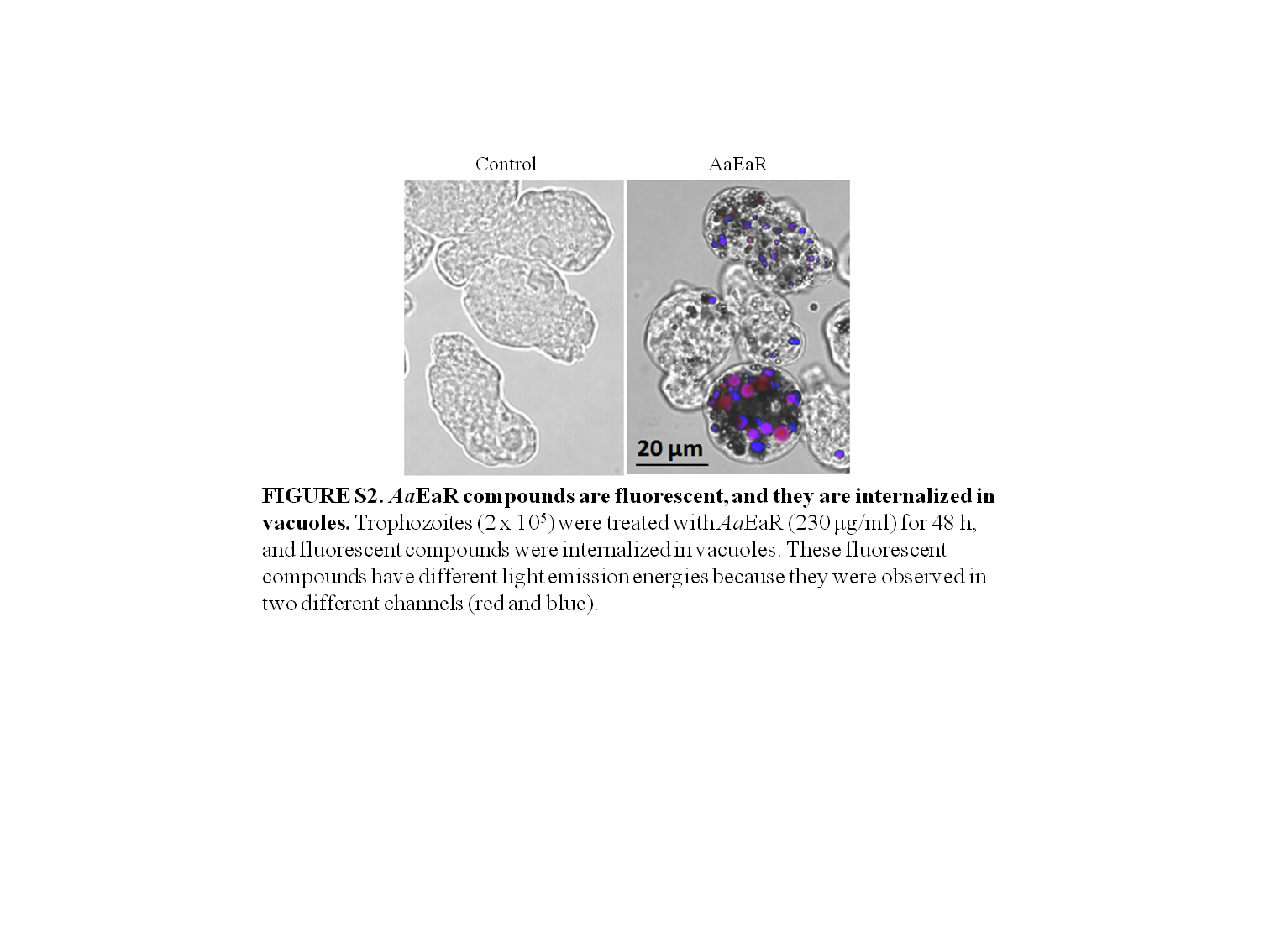

Supplement: Supplementary file 2 [file Image_2.TIF]

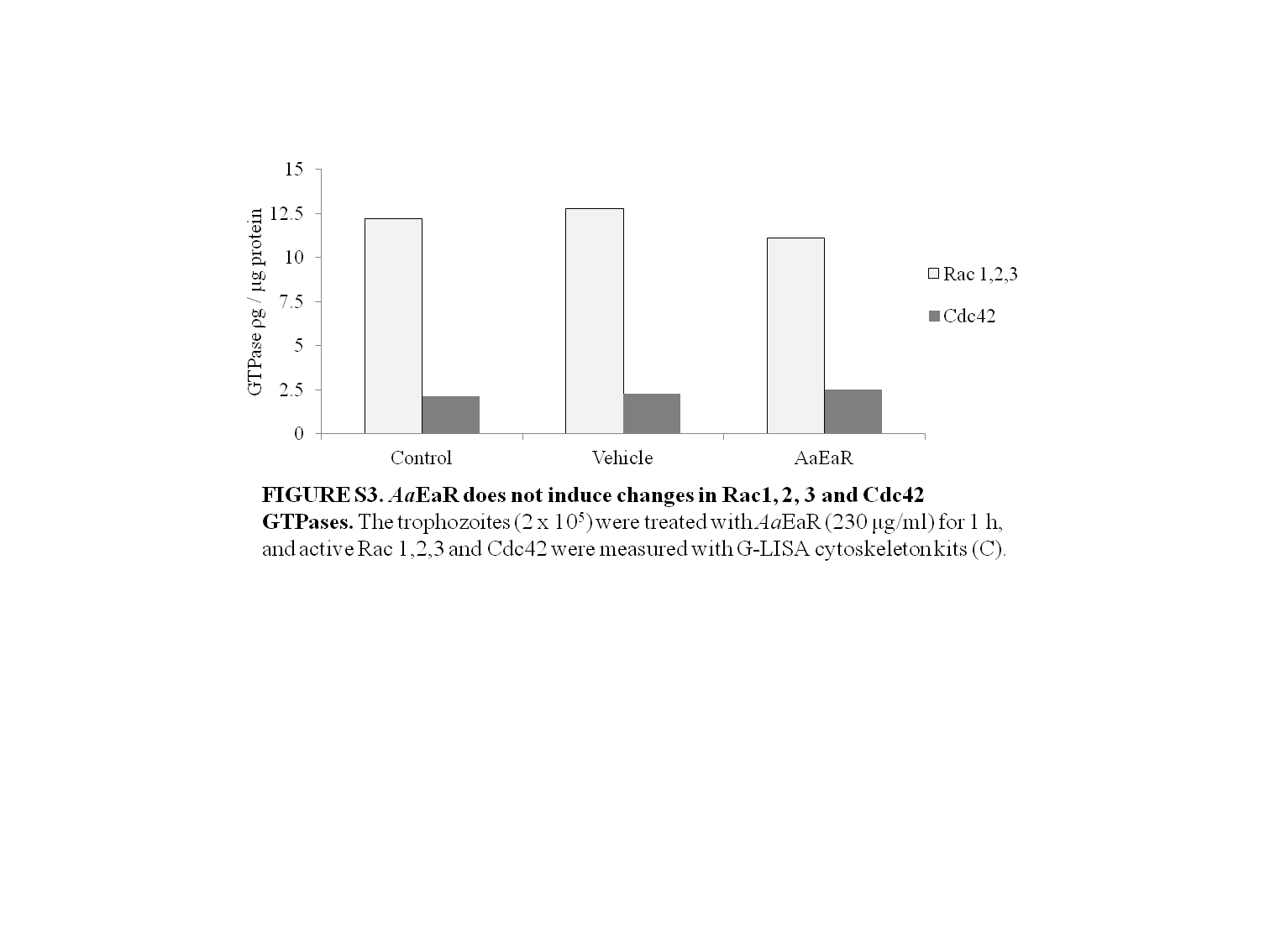

Supplement: Supplementary file 3 [file Image_3.TIF]
